# Medical Colleges

**Published:** 1860-10

**Authors:** 


### Medical Colleges.

From the many catalogues received of the various Colleges, during the present year, it is evident that the tide of Medical Students toward the Schools of the North is ebbing. This is as it should be, and as it will continue to be, until there is an equilibrium in the patronage of Medical Institutions. Medical teaching with other departments of learning, like the population of the country, commenced in the North, and it is but natural that for a time after Schools have been established in the South, and have become equally important in point of facilities for instruction in every particular, Northern Schools will be more numerously attended. The force of habit; the hold that an institution retains upon the better feelings of its alumni, making its impress through this means upon the private pupil of such graduate, and the sentiment which so generally prevails, that old established and well tried Colleges are to be preferred—all have retarded the prosperity of Southern institutions. It is not that Northern Colleges have depreciated now and become unworthy of patronage, but that Southern Schools have advanced, and should command the respect and confidence of the profession and of the learner at home. "We do not love Cæsar less, but Rome more." Again, it is not only true that the facilities for instruction in the theory of Medicine, in Southern institutions, are equal with those of any other section, but in other particulars home institutions give additional advantages. On this subject, in the Annual Announcement of the Medical Collge of Georgia, the following statement is made: "For the purpose of furnishing an education to those who expect to practice in a Southern field, where the diseases are so strikingly modified by climate and the many other influences of locality, the Trustees feel fully warranted in saying that *public opinion* has, for years past, been decidedly in favor of SOUTHERN MEDICAL INSTITUTIONS." That public opinion is now undergoing a change favorable to institutions in our midst, we have abundant evidence in the increasing number that are educated South, in proportion to the whole number of students in the Union.

We are in receipt of the Annual Announcements, for the present year, of most of the Colleges. The New Orleans Schools open the regular Course of Lectures about the 15th of November, and continue until some time in March following.

From the facilities offered by these Institutions, it would seem that nothing is wanting to make a course of study profitable in New Orleans. The increased number of students reported in their catalogues, is but another evidence of the fact. The "New Orleans School of Medicine," the advertisement of which will be found on the cover of our Journal. from its contiguity to the Charity Hospital, offers unusual advantages for clinical instruction.

"The Medical College of Alabama" also offers inducements to Medical Students, and, though young, has the prospect of a bright future. An unusually large class, for the first session, was in attendance last winter. The next Course commences about the middle of November.

From the Catalogues and Announcements of the Schools of our own State, they are prosperous, and not at all inclined to relax their efforts in future, but promise even greater facilities for learning than heretofore.

We have just received the Announcemeut of "Middle Georgia Medical College," which opens the first Session on the first Monday in November. We have had the pleasure of an intimate personal acquaintance with some of those connected with the enterprise, and hope that success may crown the effort of every laudable undertaking.

The Memphis, Nashville, Louisville, and St. Louis Schools will open their regular Winter Sessions at the usual time.—We have received the Announcement of all these energetic Western Schools, except the "University of Nashville," and we learn from other sources that it is still in a highly prosperous condition.

The Medical Department of the "University of the Pacific," San Francisco, Cal., opens its regular Course in May, which continues four months. We wish the University abundant success.

The circular of the Medical Department of "Lind University," Chicago, opens on the second Monday in October, and continues till the last of February following. The Faculty is composed of twelve Professors, arranged in two distinct departments; one for teaching the elementary or fundamental branches, the other for the practical. This is an innovation on the common system of teaching, and seems to us like real reform and improvement in Medical education. We wish Dr. Davis and his confreres success in their undertaking.

The University of Virginia commences her eight months course the first of October. Students are admitted to examination at the end of the Course without having taken lectures previously, and graduate, *provided they are found prepared for the duties of the profession.* Should the system of instruction in Lind University, and the rigid green-room exactions of the University of Virginia be adopted by Medical Colleges generally, we should have less cause to deplore the number of incompetent men in the profession.

From the circulars of the Charleston, Philadelphia and New York Medical Colleges, we see that these old, well-tried and popular Institutions are in trim for the Winter's exercises.

Other Medical Colleges, which we have not now time to notice, will be alluded to at some future time.

